# Sub-30 ms real-time, free-breathing cardiac imaging with SPIRiT

**DOI:** 10.1186/1532-429X-16-S1-W2

**Published:** 2014-01-16

**Authors:** Samuel T Ting, Yu Ding, Shivraman Giri, Ning Jin, Orlando P Simonetti, Rizwan Ahmad

**Affiliations:** 1 The Ohio State University, Columbus, Ohio, USA; 2 Siemens Healthcare, Chicago, Illinois, USA

## Background

Combined with Variable density Incoherent Spatio-Temporal Acquisition (VISTA, [1]), SPIRiT [2] reconstruction can provide an avenue for highly accelerated real-time, free-breathing cardiac imaging. Yet the high computational cost of iterative algorithms limits practical clinical implementation. The Fast Iterative Shrinkage Thresholding Algorithm (FISTA) [3] can potentially reduce the computational cost of SPIRiT [4]. In this work, we combine FISTA optimization with VISTA sampling and spatiotemporally regularized SPIRiT reconstruction to demonstrate the feasibility of real-time cine accelerated to rate 15, while providing a pathway to practical clinical application.

## Methods

Resting cine images in the short axis orientation (48 frames, 224 × 144 matrix size, 720 mm × 290 mm FOV, 8 mm slice thickness) were acquired (Siemens, Avanto 1.5T, 32 channels) under free-breathing conditions from one healthy volunteer. Data were acquired at acceleration rates ranging from R6 to R15 (temporal resolutions: 57.6 ms to 23.0 ms) using the VISTA sampling pattern. For faster processing, coil compression from 32 to 12 channels was used, and data were cropped in the frequency encoding direction to 116 pixels. The FISTA optimization algorithm was combined with spatiotemporal L1-regularization within the 3D wavelet domain to take advantage of the VISTA sampling pattern. A GRAPPA reconstruction with a 2 × 11 kernel was used as initialization for the FISTA algorithm. Both the 2 × 11 GRAPPA kernel and a 7 × 7 SPIRiT kernel were estimated from the temporal average of all frames. Acquired data were maintained fixed for the first 10% of the iterations, after which all data were allowed to be modified by the algorithm. A minimum change in the cost function was used as a stopping criterion, with truncation after 50 iterations. All data were reconstructed offline in MATLAB (version 2013b) using an Intel Core i5 workstation with 16Gb memory. SNR of reconstructed images was measured, and images were qualitatively evaluated for aliasing artifact.

## Results

**Figure 1:**
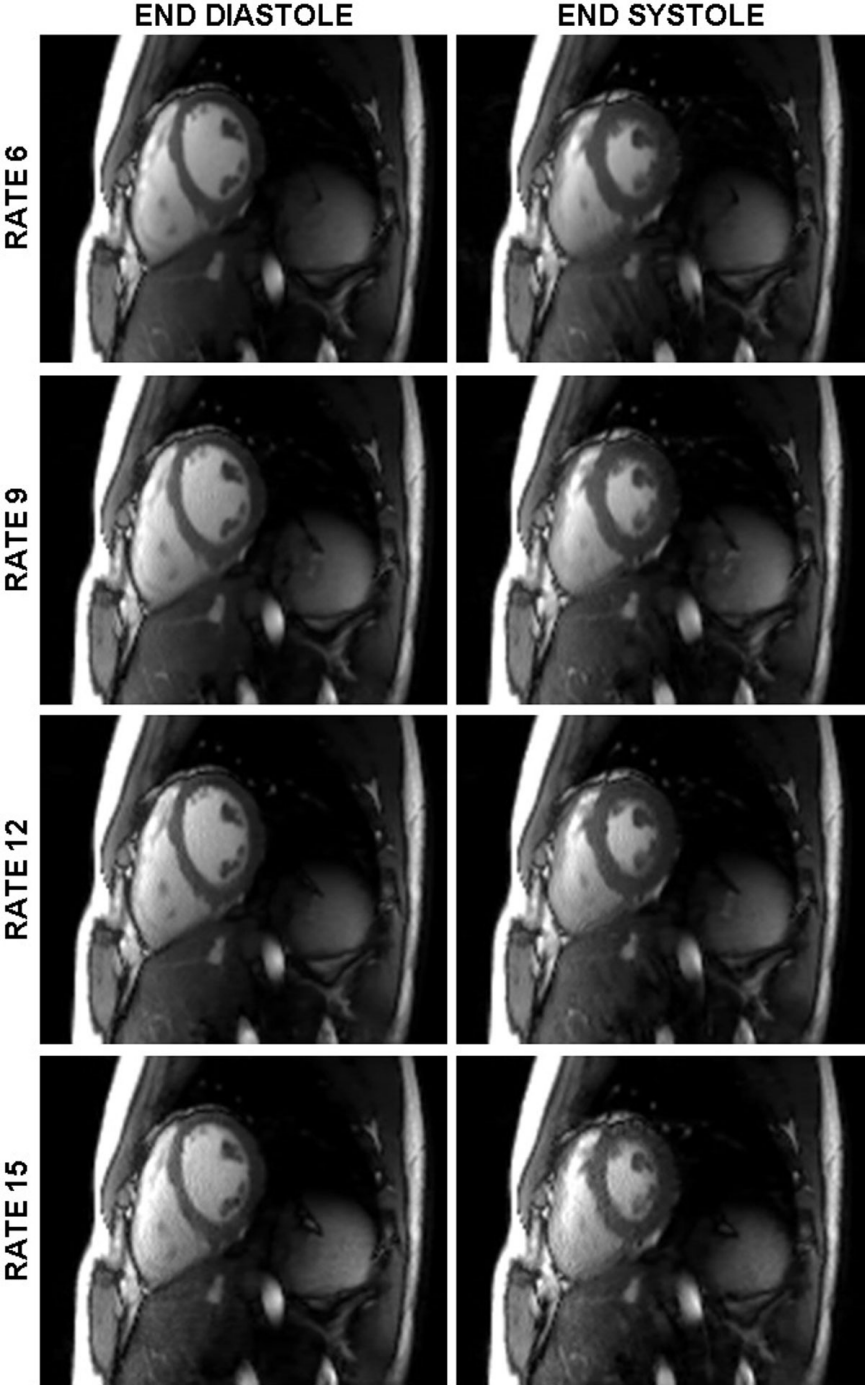
**Example frames at end diastole (left column) and end systole (right column) reconstructed at rates 6, 9, 12 and 15 using the FISTA algorithm**. The FISTA algorithm shows no appreciable loss in image quality or increase in aliasing artifact when acceleration is increased from R6 to R15.

Figure 1 shows reconstructed images at end systole and end diastole at acceleration rates 6, 9, 12, and 15. As acceleration increases from R6 to R15, high signal-to-noise is maintained without significant evidence of aliasing artifact. While reconstruction time was just under 20 minutes on a quad-core CPU system, we conservatively expect a tenfold reduction in computation time using GPU processing, allowing us to achieve the speed required for clinically practical application of this approach.

## Conclusions

Combined with VISTA and spatiotemporal regularization, the FISTA implementation of SPIRiT can provide a practical avenue for highly accelerated, real-time, free-breathing cardiac imaging.

## Funding

This work funded by NIH R01 HL102450.
